# Cardiovascular imaging research and innovation in 2023

**DOI:** 10.1093/ehjimp/qyae029

**Published:** 2024-04-18

**Authors:** Andrea Barison, Ana Teresa Timoteo, Riccardo Liga, Sonia Borodzicz-Jazdzyk, Saloua El Messaoudi, Christina Luong, Giulia Elena Mandoli, Sara Moscatelli, Arti Anushka Ramkisoensing, Sarah Moharem-Elgamal, Gianluca Pontone, Danilo Neglia

**Affiliations:** Cardiology and Cardiovascular Medicine Department, Fondazione Toscana Gabriele Monasterio, Via Moruzzi, 1, Pisa 56124, Italy; Interdisciplinary Center for Health Science, Scuola Superiore Sant'Anna, Pisa, Italy; Cardiology Department, Santa Marta Hospital, Centro Hospitalar Universitário Lisboa Central, Lisbon, Portugal; NOVA Medical School, Universidade Nova de Lisboa, Lisbon, Portugal; Department of Surgical, Medical and Molecular Pathology and Critical Care Medicine, University of Pisa, Italy; Cardiology Division, Azienda Ospedaliero-Universitaria Pisana, Pisa, Italy; Department of Cardiology, Amsterdam UMC, Vrije Universiteit Amsterdam, Amsterdam Cardiovascular Sciences, Amsterdam, The Netherlands; First Department of Cardiology, Medical University of Warsaw, Warsaw, Poland; Department of Cardiology, Radboud university medical center, Nijmegen, The Netherlands; Division of Cardiology, University of British Columbia, Vancouver, British Columbia, Canada; Department of Medical Biotechnologies, Division of Cardiology, University of Siena, Siena, Italy; Inherited Cardiovascular Diseases, Great Ormond Street Hospital, Children NHS Foundation Trust, London, UK; Institute of Cardiovascular Sciences, University College London, London, UK; Department of Cardiology, Leiden University Medical Center (LUMC), Leiden, The Netherlands; Cardiology Department, Liverpool Heart and Chest Hospital, Liverpool, UK; Department of Perioperative Cardiology and Cardiovascular Imaging, Centro Cardiologico Monzino IRCCS, Milan, Italy; Department of Biomedical, Surgical and Dental Sciences, University of Milan, Milan, Italy; Cardiology and Cardiovascular Medicine Department, Fondazione Toscana Gabriele Monasterio, Via Moruzzi, 1, Pisa 56124, Italy; Interdisciplinary Center for Health Science, Scuola Superiore Sant'Anna, Pisa, Italy

**Keywords:** echocardiography, cardiovascular magnetic resonance, computed tomography, positron emission tomography, single-photon emission tomography, multimodality imaging

## Abstract

In 2023, cardiovascular imaging has made significant advancements, in terms of technology, pathophysiology, and clinical application. In this review, the most recent research findings in the field of cardiovascular imaging are discussed. Artificial intelligence and large population cohorts, together with several technical improvements, have had a crucial impact on the technological advancements of echocardiography, cardiovascular magnetic resonance, computed tomography (CT), and nuclear medicine. In the field of ischaemic heart disease, it has been demonstrated that appropriate non-invasive imaging strategies improve patients’ management and reduce invasive procedures and the need for additional testing at follow-up. Moreover, improvements in plaque characterization with CT are an expanding field of research with relevant implications for the prediction of disease severity, evolution, and response to treatment. In the field of valvular heart disease, imaging techniques have advanced alongside improvements in transcatheter treatment for aortic stenosis, mitral, and tricuspid regurgitation. Finally, in the field of heart failure and cardiomyopathies, cardiovascular imaging has reinforced its crucial role in early diagnosis and risk evaluation, showcasing advanced techniques that outperform traditional methods in predicting adverse outcomes.

## Graphical Abstract

**Graphical Abstract:**
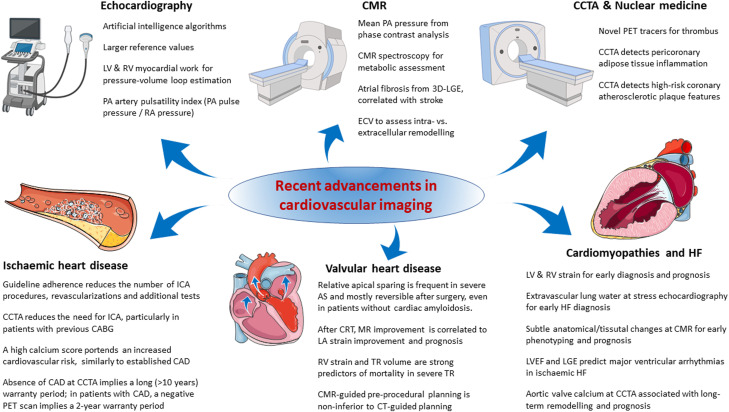
The most recent advancements of cardiovascular imaging are represented, both in terms of technological novelties for each modality (top row) and in terms of pathophysiological and clinical applications (bottom row). AS, aortic stenosis; CABG, coronary artery bypass graft; CAD, coronary artery disease; CCTA, cardiac computed tomography angiography; CMR, cardiovascular magnetic resonance; CRT, cardiac resynchronization therapy; ECV, extra-cellular volume; HF, heart failure; ICA, invasive coronary angiography; LA, left atrium; LGE, late gadolinium enhancement; LV, left ventricle; LVEF, left ventricular ejection fraction; MR, mitral regurgitation; PA, pulmonary artery; PET, positron emission tomography; RA, right atrium; RV, right ventricle; TR, tricuspid regurgitation.

## Introduction

In the past year, research in cardiovascular imaging has yielded substantial findings, providing valuable insights applicable to contemporary clinical practice. This review aims to summarize some of the most crucial research findings about technological advancements of imaging techniques and their applications in ischaemic heart disease (IHD), valvular heart disease, cardiomyopathies, and heart failure (HF).

## Technological state of the art and novelties

### Echocardiography

The application of artificial intelligence (AI) to cardiovascular imaging is evolving towards standardized acquisition and automated image interpretation. To guide deep-learning algorithms, updated normal reference values of echocardiographic parameters using recent technologies are needed.

The calculation of aortic valve area (AVA) by continuity equation, cornerstone of aortic stenosis (AS) quantification, requires a precise assessment of aortic velocity time integral (VTI), left ventricular outflow tract (LVOT) dimension, and LVOT VTI. Novel reference values across age sub-groups and genders were obtained from a large population of healthy subject of the World Alliance Societies of Echocardiography study, showing how women have smaller LVOT and AVA with dimensions increasing with age.^1^ Holste *et al*.^2^ tested AI for the detection of severe AS based only on a parasternal long-axis view, without Doppler interrogation. Developed from more than 17 500 videos, the algorithm maintained high diagnostic performance (area under the curve [AUC] > 0.94) after validation on two external cohorts, with possible useful insights for the point-of-care approach.

Updated nomograms were reported on a Norwegian healthy population of 1412 subjects (55.8% women, mean age 57.5 ± 12.4 years) for bi-dimensional and 3D echocardiographic measurements including left ventricular ejection fraction (LVEF), left ventricular (LV) and right ventricular (RV) diameters and volumes, and bi-atrial dimensions.^3^ Interestingly, both RV and left atrial (LA) measurements were slightly larger than previously reported, probably related to the different echocardiographic views. Early identification of pathological LV remodelling in young hypertensive patients can change the therapeutic approach and thus the long-term prognosis. Using a normotensive and young hypertensive data set (51.6% male, mean age 28.9 ± 5.7 years) derived from five different sites across England, Alsharqi *et al*.^4^ created a machine learning-derived echocardiographic score, testing 66 between standard and advanced (e.g. LA strain) indices, commonly used to describe cardiac remodelling. As many as 21 variables, mostly describing LA dimensions and function, LV volumes and Doppler measurements were able to justify >80% of the variance of the model and were validated on an internal cohort, showing a sensitivity of 94% and specificity of 94.6% in discriminating between normotensive and hypertensive patients.

### Cardiovascular magnetic resonance

Cardiovascular magnetic resonance (CMR) advancements relate to novel sequences, novel post-processing image analysis, and novel clinical applications. In a sub-analysis of the Clinical Evaluation of Magnetic Resonance Imaging in Coronary Heart Disease (CE-MARC) study, Swoboda *et al*.^5^ investigated the optimal method of analysis of stress CMR images. A ‘stress late gadolinium enhancement (LGE)’ analysis (which classified any stress perfusion defect as ischaemic unless corresponding to >75% infarct transmurality) had superior diagnostic accuracy to the conventional ‘stress–rest’ method, with a similar prognostic significance over a median 6.5-year follow-up. Abdula *et al*.^6^ investigated the accuracy of a novel compressed sensing accelerated phase-contrast sequence for the analysis of pulmonary artery (PA) vortex duration to estimate mean PA pressure. This sequence was able to provide a non-invasive estimation of mean PA pressure in 2-min scan time, compared to 9 min of conventional 4D flow imaging, increasing the versatility of CMR.

A prospective, randomized trial compared CMR or computed tomography (CT) guiding before transcatheter aortic valve replacement (TAVR).^7^ CMR-guided TAVR was non-inferior to CT-guided TAVR in terms of device implantation success, suggesting that CMR might become a promising alternative to CT in patients with contraindications to iodinated contrast administration. Larsen *et al*.^8^ assessed LA fibrosis in patients free of atrial fibrillation with recent ischaemic stroke (<30 days) by using a 3D LGE sequence. Patients with stroke had a larger extent of LA fibrosis than matched controls, while the extent of LA fibrosis was independent from stroke aetiology.

### Nuclear, CT, and angiography

The diagnostic accuracy of myocardial perfusion imaging (MPI) for identifying haemodynamically significant coronary artery disease (CAD) has come into debate. A sub-analysis of the PACIFIC-2 study showed that MPI with either single-photon emission tomography (SPECT), positron emission tomography (PET), or CMR had lower sensitivity (33–58%) and overall accuracy (65–72%) in detecting fraction flow reserve (FFR)-positive CAD compared with invasive coronary angiography (ICA)-derived quantitative flow ratio, suggesting that an invasive approach with ICA + quantitative flow ratio may be superior to MPI when referenced by FFR.^9^

Moreover, advancements in molecular imaging, especially with PET, have opened new avenues in clinical research. In a recent study by Tzolos *et al*.,^10^ 18F-GP1 PET, using a novel glycoprotein IIb/IIIa receptor antagonist-based radiotracer, identified intracoronary thrombus within culprit coronary arteries of acute myocardial infarction patients.

Coronary CT angiography (CCTA) is the benchmark for non-invasively assessing coronary artery structure and (peri)coronary inflammation, as demonstrated by increased CT attenuation of pericoronary adipose tissue (PCAT). Yuki *et al*.^11^ demonstrated that high PCAT attenuation correlated with a higher prevalence of vulnerable coronary plaques on optical coherence tomography.

Data on the cardiovascular effect of chest radiotherapy in breast cancer patients are quite outdated. A multimodality study by Krug *et al*. demonstrated that contemporary (low-dose) irradiation protocols did not associate with major structural and functional cardiac changes at >10 years of follow-up, including no discernible impact on CAD burden.^12^

## Ischaemic heart disease

**Figure 1:**
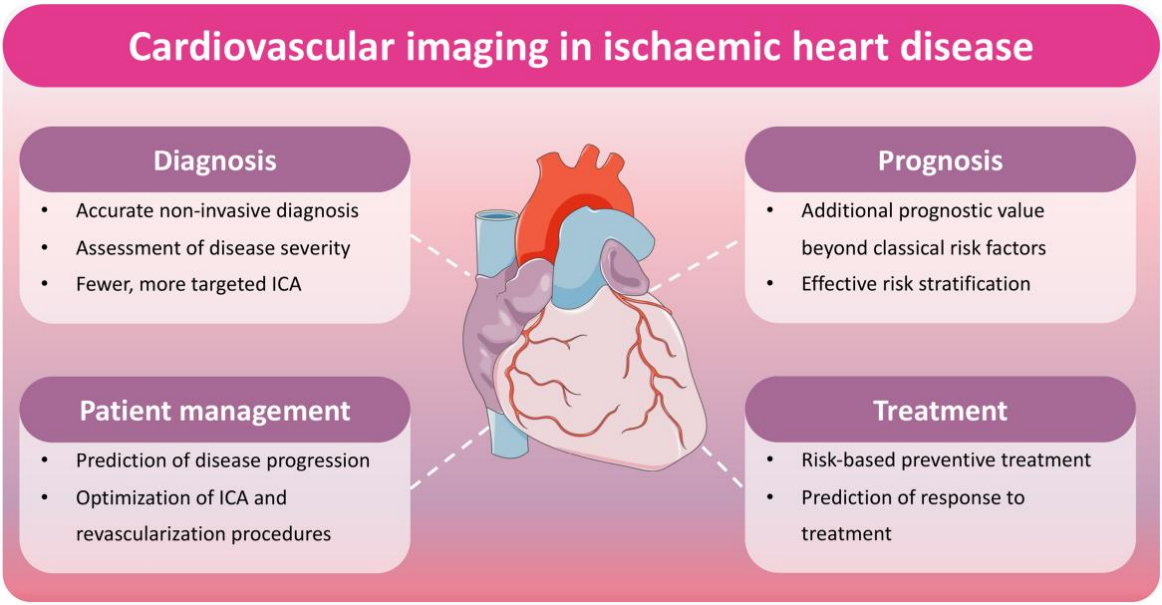
Cardiovascular imaging in IHD. The most important novelties of current imaging techniques in the clinical management of IHD are schematically indicated in the figure. ICA, invasive coronary angiography.

The use of non-invasive imaging is advocated by international guidelines to optimize the diagnostic and prognostic management of patients with IHD (*Figure 1*). The EURECA Imaging registry, a prospective international multicentre registry, evaluated the adoption of 2019 guidelines for the use of non-invasive and invasive imaging tests in chronic coronary syndromes across 5156 patients from 73 European centers.^13^ Guidelines were adopted in only 56% of patients, with the primary factors leading to non-adoption being the preference for exercise electrocardiogram over non-invasive imaging tests and the direct referral to ICA. Implementation of guidelines resulted in fewer diagnostic ICA (15% vs. 48%), fewer revascularization procedures (8% vs. 19%), higher diagnostic yield of ICA for obstructive CAD (60% vs. 39%), and higher proportion of ICA leading to coronary revascularization (54% vs. 37%). Guideline adoption also improved the quality of life, reduced the need for additional imaging tests at follow-up, and delayed late revascularization. A recent meta-analysis by Machado *et al*.^14^ showed that CCTA imaging reduces the need for ICA in a substantial proportion of patients with stable chest pain: in patients who underwent CCTA before initially scheduled ICA, only 23% underwent ICA. Patients who underwent CCTA first had also lower rates of coronary revascularization and stroke.

Several studies investigated the additional clinical value of different imaging modalities in specific IHD clinical scenarios. A recently published secondary analysis of the GadaCAD2 trial demonstrated, that in patients with suspected or known CAD, stress perfusion CMR has superior diagnostic accuracy to gated SPECT for detecting significant CAD [area under the receiver operating characteristic curve; 0.88 (0.83–0.93) vs. 0.74 (0.68–0.80), *P* < 0.001].^15^ In patients with previous coronary artery bypass graft surgery (CABG), Al Rifai *et al*.^16^ demonstrated the prognostic value of PET-derived myocardial flow reserve (<2) independent of cardiovascular risk factors and perfusion data. A randomized controlled trial by Jones *et al*.^17^ showed that incorporating adjunctive CCTA prior to ICA in patients with a history of CABG reduced procedural time, improved patient satisfaction post-ICA, lowered the incidence of contrast-induced nephropathy, and reduced the number of procedural complications and 1-year major adverse cardiovascular events (MACE).

Many studies have assessed the effectiveness of non-invasive imaging for individualized risk assessment and risk-based preventive treatment, including the potential use of CT-derived coronary artery calcium score (CACS). In a dedicated analysis from the CONFIRM registry, higher CACS was associated with increased incidence of MACE in patients without history of atherosclerotic cardiovascular disease (ASCVD).^18^ An important finding was that the highest event rates of all-cause mortality, MACEs, MACE + late revascularization, and myocardial infarction in patients with CACS > 300 were not statistically different than those observed in patients with established ASCVD. These results support the consideration of intensified medical therapy in patients with high CACS similar to patients with established ASCVD for secondary prevention. In the general population, current guidelines recommend the use of the Systematic COronary Risk Estimation 2 (SCORE2) to estimate CAD risk and determine treatment strategy. Ties *et al*. revealed, however, that SCORE2 presents limited accuracy in identifying patients with increased CACS. The miss rate was 32% and 41% for pre-screening by moderate (≥5%) SCORE2 risk and 81% and 87% for high (≥10%) SCORE2 risk, for CACS ≥ 300 and CACS ≥ 100, respectively. Conversely, the miss rate was only 8% and 11% for pre-screening by at least one CAD risk factor.^19^

Another large study focused on the ‘warranty period’, i.e. the event-free period associated with negative imaging results, helping clinicians to guide downstream management. In 2575 patients undergoing CCTA and/or stress perfusion [15O]H2O PET, Jukema *et al*. evidenced the long warranty period (>10 years) associated with a negative CTCA scan. In patients with non-obstructive or obstructive CAD at CTCA, a negative PET scan extended the warranty period of 2 years (from 5 to 7 years and from 1 to 3 years, respectively).^20^

The characterization of coronary atherosclerosis, now possible with advanced CCTA imaging, is an expanding field of research with relevant implications for the prediction of disease severity, evolution, and response to treatment. In symptomatic patients with suspected CAD, diameter stenosis ≥ 50% and necrotic core volume in CCTA were independently associated with myocardial ischaemia in [15O]H2O PET.^21^ A sub-analysis of the PARADIGM study demonstrated that in mild stenotic (25–49%) lesions with ≥2 high-risk coronary atherosclerotic plaque (HRP) features, statin therapy resulted in a 37% reduction in annual total plaque burden with decreased necrotic core volume and increased dense calcium volume. The key factors for rapid plaque progression were ≥2 HRPs, current smoking, and diabetes.^22^

PCAT attenuation is a marker of inflammation of the pericoronary fat tissue, which can be assessed by CCTA. In patients with stable CAD, Giesen *et al*. demonstrated that PCAT attenuation had weak correlation with classical markers of CAD severity but significant associations with non-calcified plaque burden and HRP features. PCAT attenuation identified high-risk patients who could benefit from more aggressive preventive therapy, beyond classical risk phenotypes.^23^

Global coronary atherosclerotic burden can be evaluated by CCTA using comprehensive scores that are able to stratify individual risk. The Leiden CCTA score was calculated in a large multicentre registry including almost 25 000 individuals (53% men) followed for 3.7 years, with the purpose of assessing possible differences according to age and sex.^24^ This study showed that coronary atherosclerosis manifests later in women, with the median risk score rising above zero 12 years later than in men (64–68 vs. 52–56 years). In both sexes, the Leiden CCTA risk score was independently associated with MACE; however, its prognostic value was definitely higher in post-menopausal women compared with men, particularly in the highest score (>20) groups (hazard ratio [HR] 6.11 and 2.25, respectively). These results underscore the need for intense medical treatment, in particular in women at risk.

## Valvular heart disease

**Figure 2:**
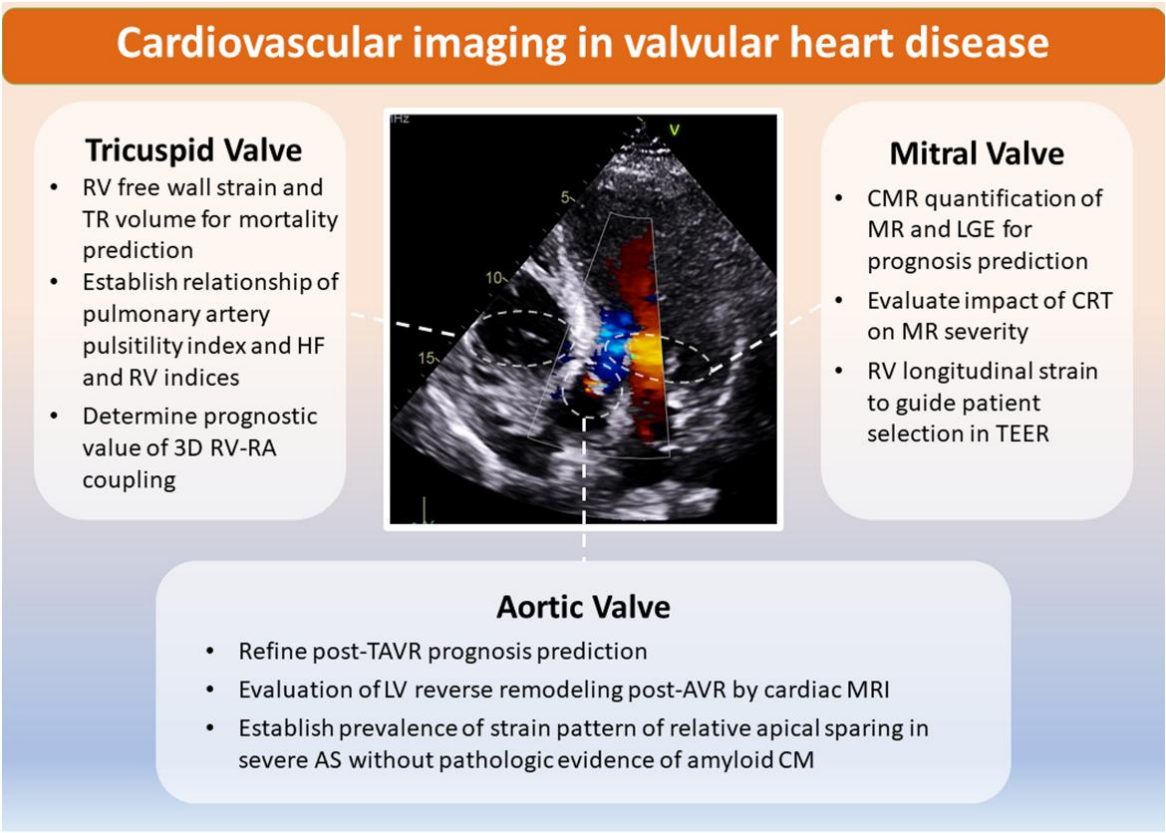
Cardiovascular imaging in valvular heart disease. The most important novelties of current imaging techniques in the clinical management of valvular heart are schematically indicated in the figure. AS, aortic stenosis; AVR, aortic valve replacement; CM, cardiomyopathy; CMR, cardiovascular magnetic resonance; CRT, cardiac resynchronization therapy; HF, heart failure; LA, left atrium; LGE, late gadolinium enhancement; LV, left ventricle; MR, mitral regurgitation; RA, right atrium; RV, right ventricle; TAVR, transcatheter aortic valve replacement; TEER, transcatheter edge-to-edge repair; TR, tricuspid regurgitation.

Over the past decade, transcatheter treatments for AS, mitral, and tricuspid regurgitation (TR) have advanced alongside improvements in imaging techniques, fuelling a rise in related research (*Figure 2*).

### Aorta

Degenerative AS, the most frequent valvular heart disease, represents about half of the publications related to valvular heart disease. Most research remains focused on surgical or transcatheter treatments; however, recent studies dwelt on the impact of AS on the myocardium, providing new insights of this disease.

LV myocardial work (MW) is a new echocardiographic-based method to assess LV function using pressure–strain loops accounting for LV afterload. Wu *et al*.^25^ analysed the usefulness of MW for the prognostic evaluation of patients undergoing TAVR. Global Work Index was independently associated with all-cause mortality and surpassed traditional LV systolic function parameters. Gutierrez-Ortiz *et al*.^26^ investigated the prognostic value of cardiac damage staging in patients undergoing TAVR. In those patients, mitral regurgitation (MR), LV global longitudinal strain (GLS), and RV-arterial coupling were independent predictors of all-cause mortality at 1 year. They introduced a new four-stage classification system, demonstrating superior predictive performance compared to existing systems. Another topic of interest is the risk of conduction abnormalities following aortic valve replacement (AVR). Laenens *et al*.^27^ found that in patients undergoing surgical AVR or TAVR, 27% developed complete bundle branch block or were implanted with a permanent pacemaker at 1 year. After adjustments, the apical-to-basal strain ratio was independently associated with conduction abnormality and could guide risk stratification in patients at risk for pacemaker implantation.

Pires *et al*.^28^ analysed 99 patients undergoing surgical AVR with CMR before and after surgery. Post-operative exams revealed reduced LV mass index and indexed extra-cellular volume (iECV) in both AS and aortic regurgitation (AR) patients, but ECV showed a slight increase only in AS patients. While AR patients developed post-operative iECV regression with stable ECV, suggesting a balanced reduction in both intra- and extra-cellular myocardial components, AS patients developed post-operative iECV regression with a slightly increased ECV, suggesting a more prominent involution of the myocardial cellular component. Abecasis *et al*.^29^ studied AS patients undergoing surgical AVR, identifying a relative apical sparing pattern (RASP) in 15.3% by pre-operative echocardiography. No suspicion of amyloid was found in pre-operative CMR and surgical septal biopsy excluded amyloidosis in all patients. Patients with RASP exhibited higher pre-operative LV mass, increased septal wall thickness, elevated N-terminal pro-B-type natriuretic peptide, lower LV ejection fraction, and higher absolute LGE mass. Follow-up at 3–6 months revealed RASP disappearance in all except two of the patients. Therefore, RASP is not specific of cardiac amyloidosis and is frequently found in severe symptomatic AS, reflecting advanced LV disease mostly reversible after surgery.

### Mitral

MR, the second most prevalent valve disease, remains a subject of substantial research, particularly concerning its impact on the myocardium. In patients with LVEF < 50%, Wang *et al*.^30^ showed that an increase in functional MR fraction was independently associated with all-cause death, heart transplant, or LV assist device implantation. Optimal threshold for moderate and severe MR were ≥20% and ≥35%, respectively, regardless of aetiology, based on the prediction of the primary outcome. Optimal LGE thresholds were ≥5% in ischaemic and ≥2% in non-ischaemic patients.

Stassen *et al*. explored the LA function response following cardiac resynchronization therapy implantation in 340 patients with at least moderate functional MR.^31^ They found that 59% had improvement in MR at 6 months. MR improvement was independently associated with an increase in LA reservoir strain, which, in turn, correlated with lower 1-year all-cause mortality.

Lupi *et al*.^32^ retrospectively studied patients with secondary MR undergoing transcatheter edge-to-edge repair. At 1-year, the best pre-procedural threshold of RV free wall longitudinal strain to predict outcome (composite of all-cause death or HF hospitalization) was −18%, whereas the best threshold of RV GLS was −15%. The identified cut-offs were independently associated with outcomes. Prognostic performance was suboptimal for other conventional echocardiographic measurements, suggesting the need for new risk stratification markers.

### Tricuspid

Until recently, diseases affecting right heart valves were often overlooked, despite the severe consequences if left untreated. In recent years, imaging techniques have advanced significantly, enabling not only the assessment of valve anatomy but also providing insights into the function and haemodynamic consequences of TR to guide treatment decisions. In severe asymptomatic TR, there is a critical need for prognostic markers to assist clinicians in treatment decisions. Akintoye *et al*.^33^ identified RV free wall strain and TR volume as strong predictors of mortality in asymptomatic patients with moderate or severe TR, with optimal thresholds of <−19% and >45 mL, respectively. Compared with symptomatic patients, mortality was lower for asymptomatic TR but increased progressively according to the number of risk factors. Kane *et al*.^34^ explored the PA pulsatility index in a large population with moderate or severe TR, revealing correlations with larger RVs, worse RV systolic function, and higher NT-pro-BNP levels. The PA pulsatility index, calculated with echocardiography as the ratio of PA pulse pressure to right atrial pressure, assesses the interaction between the PA circulation and the RV and it is correlated with invasive pulmonary pressures.

Recognizing the limitations in evaluating PA systolic pressure in patients with severe TR, Gavazzoni *et al*.^35^ explored the feasibility and association with outcomes of RV-to-PA coupling, derived from RV forward stroke volume/RV end-systolic volume measured using 3D echocardiography. This novel parameter demonstrated a stronger correlation with the composite endpoint (all-cause death and HF hospitalization) than RV ejection fraction, with a value of 0.40 identified as the best independent predictor of outcomes.

## Cardiomyopathies and myocarditis

**Figure 3:**
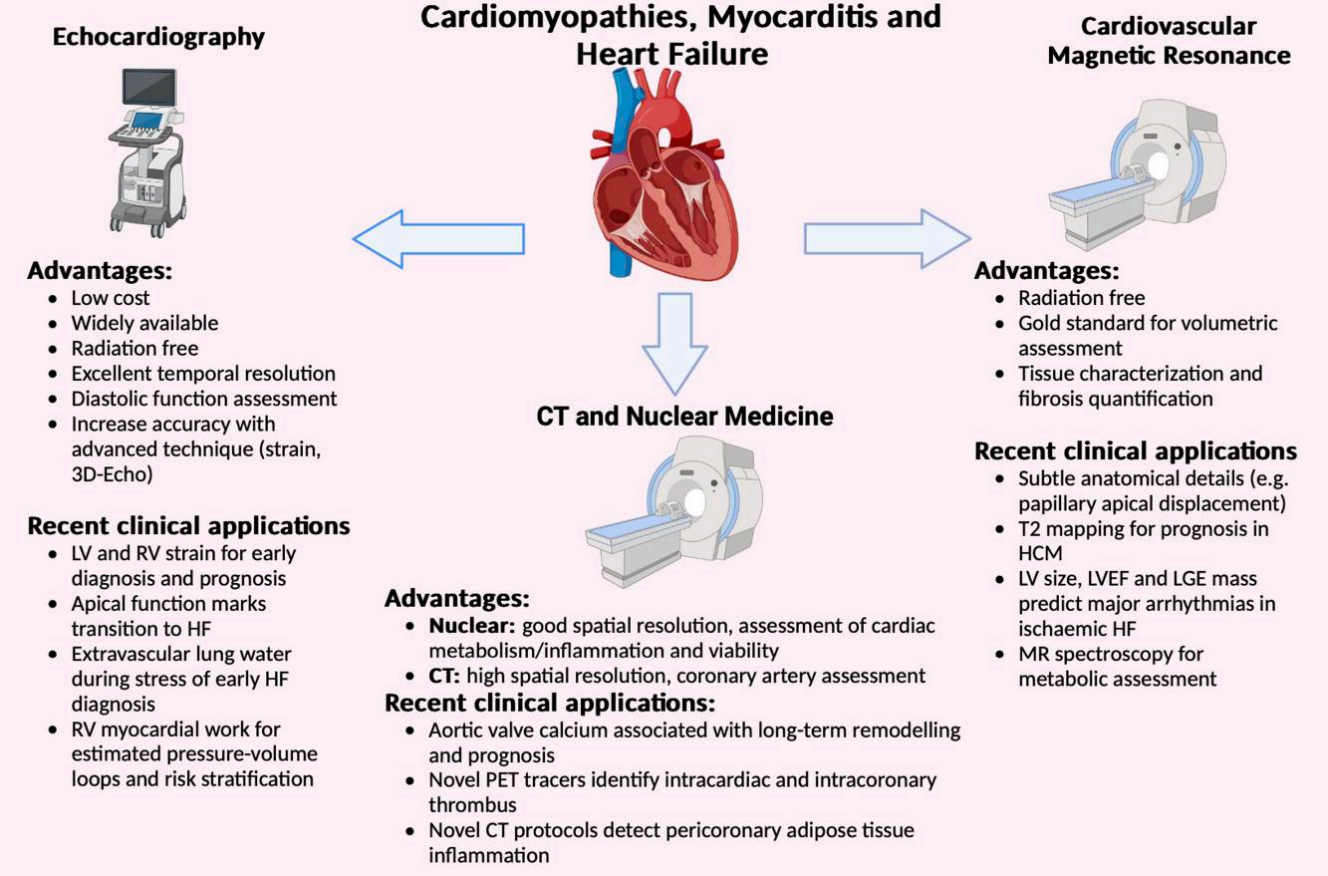
Cardiovascular imaging in cardiomyopathies, myocarditis, and heart failure. The most important advantages and the most recent advancements of each technique in the field of cardiomyopathies, myocarditis, and heart failure are schematically indicated in the figure. CT, computed tomography; CMR, cardiovascular magnetic resonance; HCM, hypertrophic cardiomyopathy; HF, heart failure; LGE, late gadolinium enhancement; LV, left ventricle; LVEF, left ventricular ejection fraction; MR, magnetic resonance; PET, positron emission tomography; RV, right ventricle.

Cardiomyopathies and myocarditis are at the forefront of cardiology's evolving landscape, with new cardiomyopathy guidelines released in 2023 and myocarditis guidelines expected for 2025. These developments emphasize the vital role of cardiovascular imaging in early diagnosis and risk evaluation, showcasing advanced techniques that outperform traditional methods in predicting adverse outcomes (*Figure 3*). Specifically, myocardial strain assessment is gaining attention for its ability to detect subclinical biventricular dysfunction. Research by Namasivayam *et al*.^36^ on arrhythmogenic RV cardiomyopathy (ARVC) revealed that RV strain assessment surpasses existing echocardiographic standards, uncovering previously undetected ARVC cases. Although LV strain assessment did not enhance diagnostic precision, it was instrumental in identifying high-risk patients. Instead, in the context of hypertrophic cardiomyopathy (HCM), Choi *et al*.^37^ found that global LV GLS crucially predicts cardiovascular risks, such as sudden cardiac death (SCD) or SCD-equivalent events, in patients with low to normal EF. Moreover, 3D strain echocardiography has emerged as a sophisticated method, with the study of Marek *et al*.^38^ demonstrating its correlation with HF severity and long-term prognosis in Fabry disease, a phenocopy of HCM. Additionally, reduced posterolateral 3D circumferential strain highlights the characteristic posterolateral scarring observed in Fabry patients. Akintoye *et al*.^39^ further explored the utility LA strain in predicting thromboembolism independently of atrial fibrillation presence in cardiac amyloidosis.

CMR is crucial for prognosis, particularly through its ability to characterize myocardial tissue. In HCM patients, high T2 mapping values were associated with a worse prognosis on top of established risk factors, including extensive LGE.^40^ Filomena *et al*.^41^ demonstrated that papillary muscle displacement is an aspect of apical HCM and may even precede the onset of hypertrophy, highlighting the importance of structural features in early disease detection and diagnosis.

## Heart failure

Within the field of HF, Hundertmark *et al*.^42^ found that sodium–glucose co-transporter 2 inhibitors improve energy production without affecting the phosphocreatine:ATP ratio at magnetic resonance spectroscopy. Zhu *et al*.^43^ showed that the burden of aortic valve calcium is associated with adverse cardiac remodelling and increased risk of *de novo* HF at 10-year follow-up. Biering-Sørensen *et al*.^44^ showed that apical mechanical function is preserved in hypertensive heart disease while it was impaired in HF with preserved ejection fraction (HFPEF) and may contribute to the transition from asymptomatic to symptomatic heart disease. Kagami *et al*.^45^ found that an increase in extravascular lung water during exercise, as indicated by the presence of ultrasound B-lines, is mostly present during the recovery period and improves the diagnosis of HFPEF. The DERIVATE (CarDiac MagnEtic Resonance for Primary Prevention Implantable CardioVerter DebrillAtor ThErapy)-ICM registry is an international, multicentre study that assessed the additional prognostic value of CMR in patients with ischaemic cardiomyopathy and LVEF < 50%: during a median 3-year follow-up, LV end-diastolic volume, LVEF and LGE mass were independent predictors of arrhythmic events. Compared with the echocardiographic-based cut-off of 35%, a CMR risk score identified a subset of patients with a LVEF ≤ 35% at low risk and also a group of patients at high risk despite a LVEF ≥ 35% with a net reclassification improvement of 31.7%.^46^ Landra *et al*.^47^ assessed RV MW in patients undergoing LV assist device implantation: a lower RV global work efficiency was associated with the occurrence of early (<30 days) RV failure and with increased mortality at 1-year follow-up.

## Learning points

The recent advancements in cardiovascular imaging collectively underscore the transformative impact of cutting-edge imaging and structural analysis in cardiology, setting new standards for the early detection, prognosis, and tailored treatments across the whole spectrum of heart diseases (*Graphical abstract*).

## Lead author biography


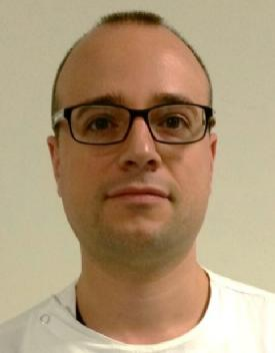


Dr Andrea Barison graduated in Medicine at the University of Pisa and the Scuola Superiore Sant'Anna in 2005, specialized in Cardiology in 2009 and obtained a PhD in Translational Medicine in 2013. He is a consultant cardiologist at the Fondazione Toscana ‘Gabriele Monasterio’ and an affiliate researcher at the Scuola Superiore Sant'Anna in Pisa (Italy). His clinical and research activities comprise cardiomyopathies and cardiac magnetic resonance. He is member of the Society for Cardiovascular Magnetic Resonance (SCMR), the European Society of Cardiology (ESC), the European Association for Cardiovascular Imaging (EACVI), and the Italian Society of Cardiology (SIC).

## Data Availability

No new data were generated or analysed in support of this research.
